# Metformin Protects Against Cisplatin-Induced Tubular Cell Apoptosis and Acute Kidney Injury via AMPKα-regulated Autophagy Induction

**DOI:** 10.1038/srep23975

**Published:** 2016-04-07

**Authors:** Jianzhong Li, Yuan Gui, Jiafa Ren, Xin Liu, Ye Feng, Zhifeng Zeng, Weichun He, Junwei Yang, Chunsun Dai

**Affiliations:** 1 Center for Kidney Diseases, 2nd Affiliated Hospital, Nanjing Medical University, 262 North Zhongshan Road, Nanjing, Jiangsu, P. R. China

## Abstract

Metformin, one of the most common prescriptions for patients with type 2 diabetes, is reported to protect the kidney from gentamicin-induced nephrotoxicity. However, the role and mechanisms for metformin in preventing cisplatin-induced nephrotoxicity remains largely unknown. In this study, a single intraperitoneal injection of cisplatin was employed to induce acute kidney injury (AKI) in CD1 mice. The mice exhibited severe kidney dysfunction and histological damage at day 2 after cisplatin injection. Pretreatment of metformin could markedly attenuate cisplatin-induced acute kidney injury, tubular cell apoptosis and inflammatory cell accumulation in the kidneys. Additionally, pretreatment of metformin could enhance both AMPKα phosphorylation and autophagy induction in the kidneys after cisplatin injection. In cultured NRK-52E cells, a rat kidney tubular cell line, metformin could stimulate AMPKα phosphorylation, induce autophagy and inhibit cisplatin-induced cell apoptosis. Blockade of either AMPKα activation or autophagy induction could largely abolish the protective effect of metformin in cisplatin-induced cell death. Together, this study demonstrated that metformin may protect against cisplatin-induced tubular cell apoptosis and AKI through stimulating AMPKα activation and autophagy induction in the tubular cells.

Cisplatin-based chemotherapeutic strategy has been clinically used for decades in patients suffered from several types of solid tumor such as non-small cell lung cancer and prostate cancer^1^. Unfortunately, approximate 25–30% of the patients treated with cisplatin may develop nephrotoxicity such as acute kidney injury (AKI). Except for the supportive regimens including fluid resuscitation and renal replacement therapy, there is no specific therapeutic strategy available currently for alleviating AKI in patients^2^. Hence, identifying the new agents for ameliorating cisplatin-induced acute kidney injury may benefit the patients who require cisplatin-based chemotherapy.

Cisplatin or its metabolites may be absorbed by kidney tubular cells through organic cation transporters (OCT) located on the basolateral side of the tubular cells, which will lead to subsequent tubular cell death and AKI^3^. Since tubular cell death including apoptosis and necrosis is the precipitating factor for cisplatin-induced AKI in both patients and animal models^2^,^4^, protecting tubular epithelial cells from death should be effective in halting the initiation and progression of cisplatin-induced nephotoxicity^5^,^6^.

Accumulated evidences demonstrated that autophagy, characterized by part of the cytoplasm, organelles, or membrane engulfed by a double-membrane structure and targeted for destruction in lysosomes^7^, may protect against cisplatin-induced tubular cell death^8^,^9^,^10^,^11^,^12^,^13^,^14^. It has been reported that mTOR signaling may regulate autophagy induction and affect tubular cell death through different mechanisms^14^,^15^,^16^,^17^. Except for regulating cell growth, mitochondrial biogenesis, oxidative stress, cell polarity and migration^18^,^19^, AMP-activated protein kinase (AMPK) activation may inhibit mammalian target of rapamycin complex 1 (mTORC1) signaling pathway and stimulate autophagy in many cell types^18^,^20^,^21^,^22^,^23^. In a mouse model with kidney ischemia-reperfusion injury, activation of AMPK with AICAR or metformin could mitigate the tubular cell injury^24^.

Metformin, one of the most common prescriptions for the patients with type 2 diabetes^25^,^26^,^27^, may reduce cancer risk and suppress tumourigenesis through AMPK-dependent suppression of the mammalian target of rapamycin (mTOR) pathway^28^,^29^,^30^,^31^,^32^,^33^,^34^. Metformin can also alleviate pain and the loss of tactile function in a mouse model of chemotherapy-induced peripheral neuropathy^35^. Additionally, our previous study demonstrated that metformin may inhibit cell apoptosis via autophagy induction in cultured tubular cells^14^. Thus, it is highly possible that metformin may protect against cisplatin-induced nephrotoxicity via activating AMPK and autophagy in tubular epithelial cells.

Here, we found that metformin could ameliorate cisplatin-induced tubular cell apoptosis in cultured NRK-52E cells and AKI in mice. Metformin could stimulate AMPKα phosphorylation and autophagy induction in cultured NRK-52E cells. Blockade of autophagy or AMPKα activation could largely diminish the protective effect for metformin in cisplatin-induced tubular cell death. This study suggests that metformin may protect against cisplatin induced tubular cell apoptosis and AKI through stimulating AMPKα activation and autophagy induction.

## Results

### Metformin protects against cisplatin-induced AKI

**Figure 1:**
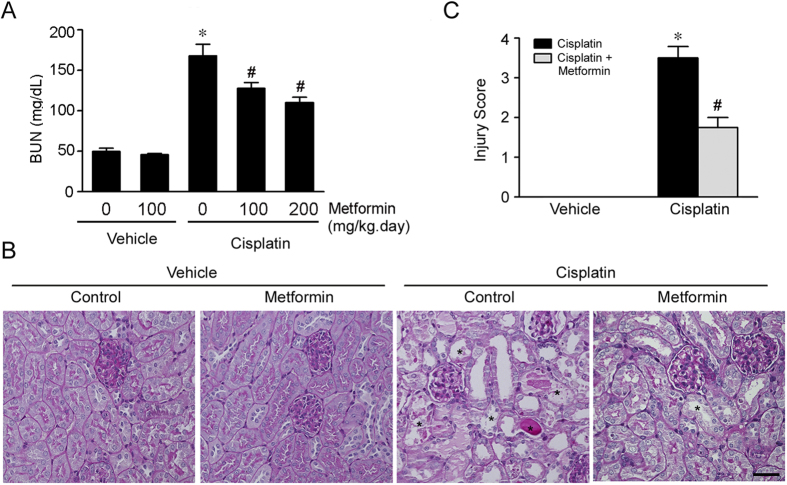
Metformin protects against cisplatin-induced AKI. The male CD1 mice were treated with vehicle or metformin (100 or 200 mg/kg per day, ip) for 3 days, followed by cisplatin injection (20 mg/kg, ip), and sacrificed at day 2 after cisplatin injection. (**A**) The graph showing the blood urea nitrogen (BUN) levels in each group. *P < 0.05 compared to vehicle control (n = 6 − 18); ^#^*P* < 0.05 compared to mice treated with cisplatin alone (n = 6 − 18). (**B**) Periodic acid-Schiff (PAS) staining showing the histological changes within groups. The asterisks indicate the injured tubule. Bar = 50 μm. (**C**) The graph showing the injury scores for kidney damage within groups. *P < 0.05 compared to vehicle control (n = 3 − 5); ^#^*P* < 0.05 compared to mice treated with cisplatin alone (n = 5).

Male CD1 mice were intraperitonially injected with cisplatin at 20 mg/kg to induce acute kidney injury as previous reported^14^. The mice developed severe acute kidney dysfunction exhibited as elevated BUN level at day 2 after cisplatin injection (Fig. 1A). To determine the role of metformin on cisplatin-induced AKI, the mice were pretreated with metformin at different dosage (100 mg/kg.d and 200 mg/kg.d) three days before cisplatin injection. At day 2 after cisplatin injection, comparing to the mice received cisplatin alone, metformin could significantly attenuate cisplatin-induced kidney dysfunction at a dose-dependent manner (Fig. 1A). PAS staining revealed that the mice developed severe kidney histological abnormalities including tubular cell death, tubular cell detachment and cast formation at day 2 after cisplatin injection, whereas kidney damage was largely diminished in mice pretreated with metformin (Fig. 1B,C). Together, these results suggest that metformin may protect against cisplatin-induced AKI in mice.

### Metformin reduces tubular cell apoptosis in the kidneys with cisplatin-induced AKI

**Figure 2:**
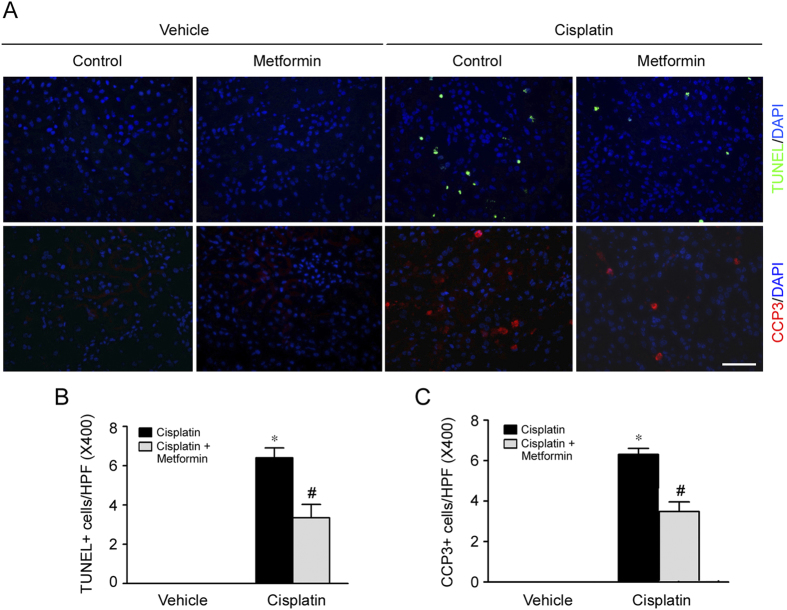
Metformin reduces tubular cell apoptosis in the kidneys with cisplatin-induced AKI. (**A**) Representative micrographs showing the TUNEL staining and immnuo-staining images for cleaved caspase 3 among different groups. Bar = 50 μm. (**B**) Quantitative analysis for the number of TUNEL staining positive cells among different groups. *P < 0.05 compared to vehicle control, (n = 3 − 5); ^#^*P* < 0.05 compared to mice treated with cisplatin alone (n = 5). (**C**) Quantitative determination for the number of cleaved caspase 3 positive cells among different groups. *P < 0.05 compared to vehicle control (n = 3 − 5); ^#^*P* < 0.05 compared to mice treated with cisplatin alone (n = 5).

Tubular cell apoptosis plays an essential pathogenic role for cisplatin-induced AKI. In this study, cell apoptosis was examined by terminal deoxynucleotidyl transferase-mediated digoxigenin-deoxyuridine nick-end labeling (TUNEL) staining and immuno-staining for cleaved caspase 3 in kidney tissues. As shown in Fig. 2, few apoptotic cells were detected in the kidney tissues from control or metformin treated mice. However, at day 2 after cisplatin injection, the number of apoptotic cells in the kidney tissues exhibited as TUNEL staining positive or cleaved caspase 3 immuno-staining positive was significantly increased, while cell apoptosis was much less in mice pretreated with metformin. These results suggest that metformin pretreatment can mitigate cisplatin-induced cell apoptosis in mice.

### Metformin decreases inflammatory cell accumulation in the kidneys with cisplatin-induced AKI

**Figure 3:**
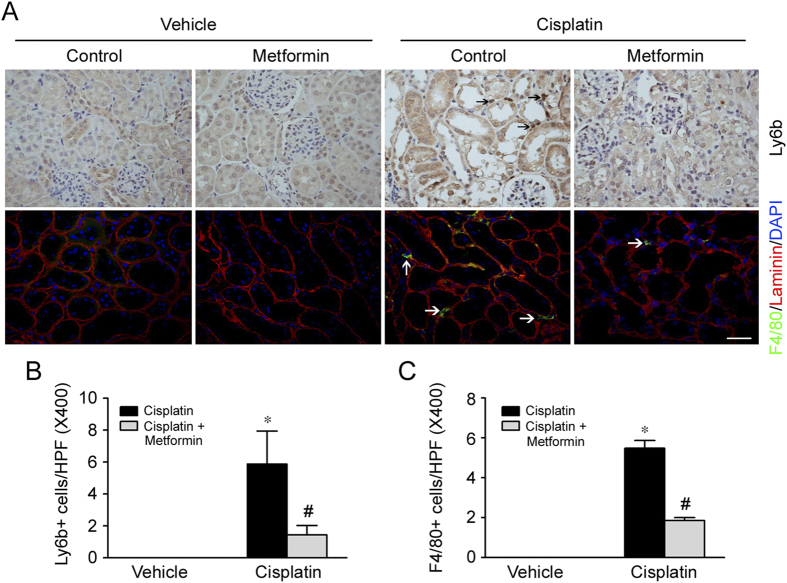
Metformin diminishes inflammatory cell accumulation in the kidneys with cisplatin-induced AKI. (**A**) Representative micrographs showing the immuno-staining images for Ly6b and F4/80 among different groups. Arrows indicate Ly6b or F4/80 staining positive cells. Bar = 50 μm. (**B**) Quantitative determination of Ly6b-positive cells within kidney tissues among different groups. *P < 0.05 compared to vehicle control (n = 3 − 5); ^#^*P* < 0.05 compared to mice treated with cisplatin alone (n = 5). (**C**) Quantitative determination of F4/80-positive cells within kidney tissues among different groups. *P < 0.05 compared to vehicle control (n = 3 − 5); ^#^*P* < 0.05 compared to mice treated with cisplatin alone (n = 5).

Besides cell apoptosis, kidney interstitial inflammation also plays a critical role for cisplatin-induced nephrotoxicity. To assess the role for metformin on inflammatory cell accumulation in kidneys after cisplatin injection, the kidney tissues were stained with antibody against ly6b or F4/80 to identify neutrophils or macrophages, respectively. As shown in Fig. 3, at day 2 after cisplatin injection, comparing to the control mice, neutrophil and macrophage accumulation were largely increased in the kidney tissues with cisplatin-induced AKI. Whereas inflammatory cell accumulation was much less in the kidneys from mice treated with metformin plus cisplatin compared to the mice treated with cisplatin alone.

### Administration of Metformin enhances AMPKα phosphorylation and autophagy induction in the kidneys from mice treated with cisplatin

**Figure 4:**
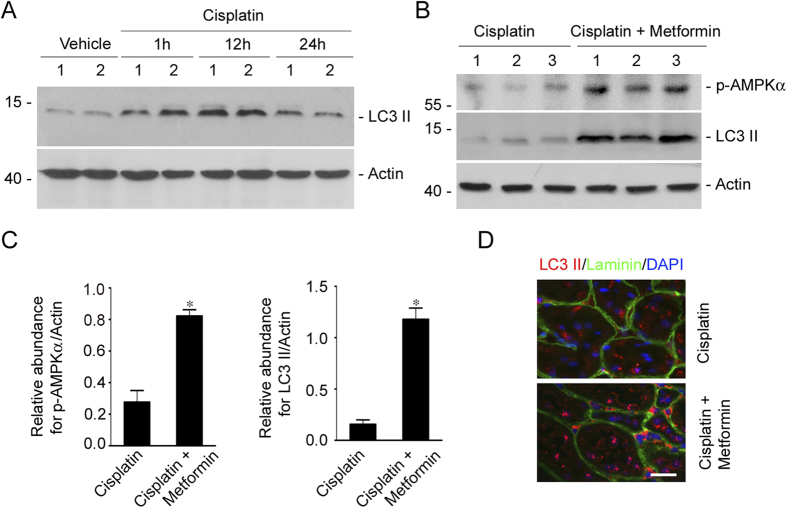
Metformin promotes AMPKα activation and autophagy induction in the kidneys with cisplatin-induced AKI. (**A**) Western blot assay showing the induction of LC3-II in the mouse kidneys after cisplatin injection. CD1 mice were injected with cisplatin (20 mg/kg) intraperitoneally, and the kidneys were collected at 1, 12 and 24 hours after injection. Numbers indicate the individual animal within each group. The gels were run under the same experimental conditions. (**B**) Western blot assay showing the abundance of p-AMPKα and LC3-II in cisplatin or cisplatin plus metformin treated kidneys. Numbers indicate the individual animal within each group. (**C**) The graph showing the results of semiquantitative analysis for p-AMPKα and LC3-II protein abundance among groups. *P < 0.05 compared to cisplatin treated cells (n = 3). The gels were run under the same experimental conditions. (**D**) Representative micrographs showing the immunofluorescent staining images for LC3β in kidneys. The intensive dot-like LC3β puncta were much more in the mice treated with metformin plus cisplatin comparing to those treated with cisplatin alone. Kidney sections were count stained with DAPI to visualize the nuclei. Bar = 20 μm.

Metformin is able to induce autophagy via activating AMPK in cardiocytes and tumor cells. To further investigate the underlying mechanisms for metformin in protecting against cisplatin-induced AKI, we detected the AMPKα activation and autophagy in the mouse kidneys. At first, CD1 mice were injected intraperitoneally with cisplatin and sacrificed at 1, 12 and 24 hours after injection. Western blotting assay revealed that LC3-II protein abundance was increased at 1 hr and peaked at 12 hrs in the kidneys after cisplatin injection, suggesting the induction of autophagy (Fig. 4A). Then, CD1 mice were pretreated with metformin (100 mg.kg.day) at 3 days before cisplatin injection. The mice were sacrificed at 12 hrs after cisplatin injection. Western blotting assay showed that p-AMPKα as well as LC3-II were significantly increased in the kidney lysates from the mice treated with metformin plus cisplatin compared to the mice treated with cisplatin alone (Fig. 4B,C). Immunofluorescent staining for LC3β showed that a few intensive dot-like LC3β puncta appear in tubular cells from mice treated with cisplatin alone, whereas the puncta were much more in the mice treated with metformin plus cisplatin, suggesting the enhancement of autophagy by metformin treatment (Fig. 4D). These results indicate that metformin may exacerbate AMPKα activation and autophagy induction in the kidneys with cisplatin-induced AKI.

### Metformin attenuates tubular cell apoptosis stimulated by cisplatin in NRK-52E cells

**Figure 5:**
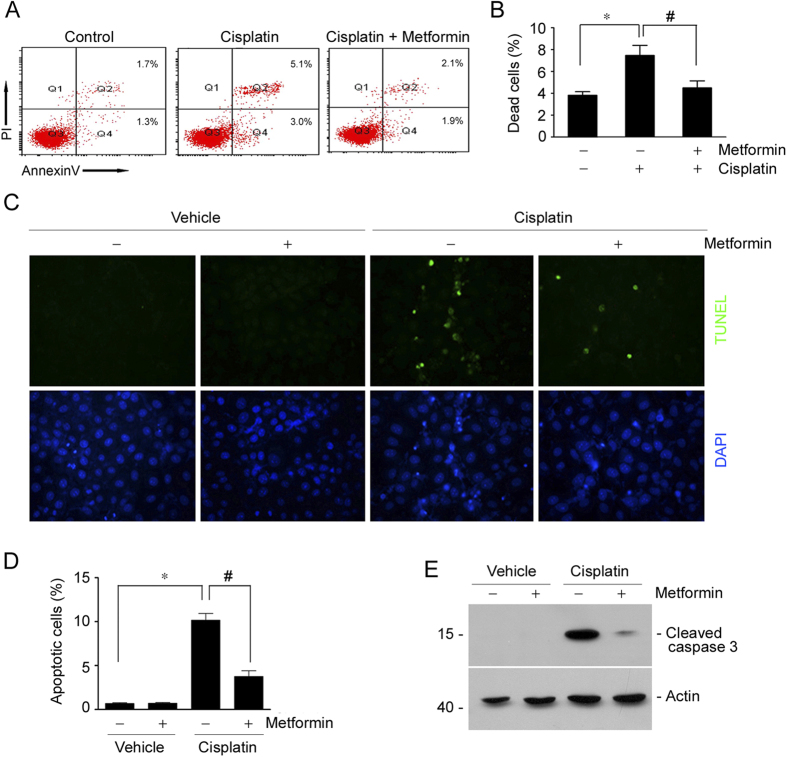
Metformin prevents cisplatin-induced cell apoptosis in cultured NK-52E cells. (**A**) The representative graphs showing the results of FACS analysis. NRK-52E cells treated with cisplatin w/o metformin were stained with annexin-V-FITC (20 mg/ml) and PI (20 mg/l) to identify cell apoptosis. (**B**) The graph showing the cell apoptosis among different groups. *P < 0.05 compared to control (n = 4); ^#^*P* < 0.05 compared to cells treated with cisplatin alone (n = 4). (**C**) Representative micrographs showing TUNEL staining among different groups as indicated. (**D**) The graph showing the quantitative determination of TUNEL staining positive cells among different groups. Data are presented as the percentage of the TUNEL staining positive cells. *P < 0.05 compared to vehicle control (n = 3); ^#^*P* < 0.05 compared to mice treated with cisplatin alone (n = 3). (**E**) Western blot assay showing the abundance of cleaved caspase 3 among different groups. The gels were run under the same experimental conditions.

To explore the role for metformin in protecting against cisplatin-induced tubular cell death in cultured cells, we treated NRK-52E cells, a rat kidney tubular epithelial cell line, with metformin, followed by cisplatin administration to induce cell death. At 12 hrs after cisplatin treatment, the cells were harvested, costained with annexin V/PI and analyzed by FACS analysis to identify apoptotic cells. As shown in Fig. 5A,B, flow cytometric analysis revealed that the percentage for dead cells including early cell apoptosis (annexin V^+^/PI^−^) and late cell apoptosis (annexin V^+^/PI^+^) was markedly increased after cisplatin treatment, while pre-incubation of metformin could significantly diminish cisplatin-triggered cell apoptosis. We also performed TUNEL staining on these cells. The results showed that the number of TUNEL staining positive cells was largely decreased in metformin pretreated cells compared to those treated with cisplatin alone (Fig. 5C,D). Consistently, western blot results showed that cisplatin could induce caspase 3 cleavage, while metformin largely diminished it (Fig. 5E). Together, it is clear that metformin may directly inhibit cisplatin-induced tubular cell apoptosis in cultural NRK-52E cells.

### Metformin activates AMPKα and induces autophagy in cultured NRK-52E cells

**Figure 6:**
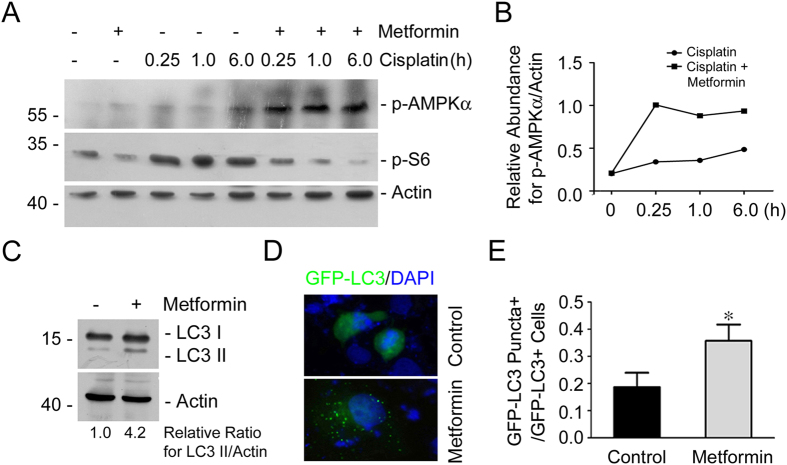
Metformin promotes AMPKα activation and autophagy induction in NRK-52E cells. (**A**,**B**) Western blot analysis showing the abundance of p-AMPKα and p-S6 at different time points after cisplatin treatment in NRK-52E cells. The antibody against actin was probed as normalization. The gels were run under the same experimental conditions. (**C**) Western blot analysis showing the induction of LC3-II abundance in NRK-52E cells treated with metformin plus cisplatin. The gels were run under the same experimental conditions. (**D**) Representative micrographs showing the autophagosome formation in NRK-52E cells after metformin treatment. NRK-52E cells were transiently transfected with GFP-LC3 expression plasmid for 24 h, followed by metformin treatment for 12 h. The transfected cells with dot-like GFP-LC3 puncta were considered as autophagosome formation in the cells. (**E**) The graph showing the quantitative analysis for autophagosome formation in NRK-52E cells after metformin treatment. Data are presented as the percentage of the GFP-LC3 puncta positive cells. *P < 0.05 compared to the control cells (n = 3).

To further clarify the underlying mechanisms for metformin in mitigating cisplatin-induced tubular cell apoptosis, the role of metformin on AMPK signaling and autophagy induction was examined in NRK-52E cells. Western blot analysis showed that the abundance of phosphorylated AMPKα was increased slightly in the cells after cisplatin treatment, whereas pretreatment of metformin could further enhance it. Additionally, phosphorylated S6, an mTORC1 signaling molecule, was increased at as early as 15 min after cisplatin treatment, while pretreatment of metformin could markedly decrease it, suggesting the inhibition of mTORC1 signaling (Fig. 6A,B). Western blotting assay showed that LC3-II was largely induced in NRK-52E cells treated with metformin, suggesting the induction of autophagy in NRK-52E cells (Fig. 6C). NRK-52E cells were transiently transfected with GFP-LC3 plasmid and then treated with metformin for 12 hours. GFP-LC3 was diffusely distributed throughout the cell cytosol in the control cells, while the number of cells with green intense dot-like GFP-LC3 puncta was significantly increased in cultured cells treated with metformin, indicating the induction for the formation of autophagosomes in metformin-treated cells (Fig. 6D,E). Thus, these results suggest that metformin may activate AMPKα and induce autophagy in NRK-52E cells.

### Inhibition of autophagy by 3-methyladenine abolishes the protective effect for metformin in cisplatin-induced cell apoptosis in NRK-52E cells

**Figure 7:**
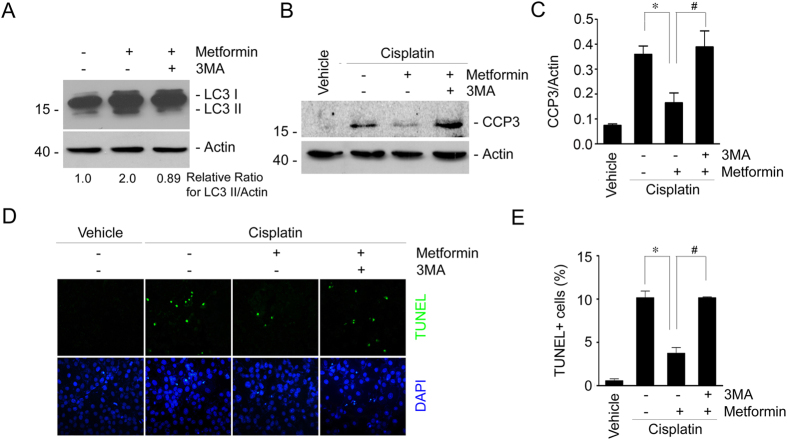
Inhibition of autophagy by 3-methyladenine diminishes the protective effect for metformin in cisplatin-induced cell apoptosis in NRK-52E cells. (**A**) Western blot assay showing the LC3-II abundance in NRK-52E cells. NRK-52E cells were incubated with metformin in the absence or presence of 3MA (10 mM) for 12 h. The gels were run under the same experimental conditions. (**B**) Western blot assay showing caspase 3 cleavage in NRK-52E cells. The gels were run under the same experimental conditions. (**C**) The graphs showing the semi-quantitative analysis results for the abundance of cleaved caspase 3 protein in NRK-2E cells. *P < 0.05 compared to the cells treated with cisplatin alone (n = 4); ^#^*P* < 0.05 compared to the cells treated with cisplatin plus metformin (n = 4). (**D**) Representative micrographs showing the TUNEL staining among different groups as indicated. (**E**) The graphs showing the quantitative determination of TUNEL-positive cells among different groups. Data are presented as the percentage of the TUNEL-positive cells. *P < 0.05 compared to the cells treated with cisplatin alone (n = 3); ^#^*P* < 0.05 compared to the cells treated with cisplatin plus metformin (n = 3).

To further determine the role of autophagy induction in mediating the protective effect for metformin in cisplatin-induced tubular cell death, we used 3-methyladenine (3-MA) to suppress autophagy in NRK-52E cells. Western blotting assay showed that metformin could induce LC3-II formation. As expected, 3-MA could largely abolish LC3-II induction (Fig. 7A). We then detected cleaved caspase 3 in NRK-52E cells with western blotting assay and found that cisplatin-induced caspase 3 cleavage was largely abolished by metformin treatment, whereas 3MA could restore the reduction of caspase 3 cleavage in NRK-52E cells treated with metformin plus cisplatin (Fig. 7B,C). TUNEL staining further confirmed the results of western blotting assay (Fig. 7D,E). These results suggest that autophagy induction is required for metformin in protecting against cisplatin-stimulated tubular cell apoptosis.

### Down regulating AMPKα expression diminishes metformin-promoted tubular cell survival

**Figure 8:**
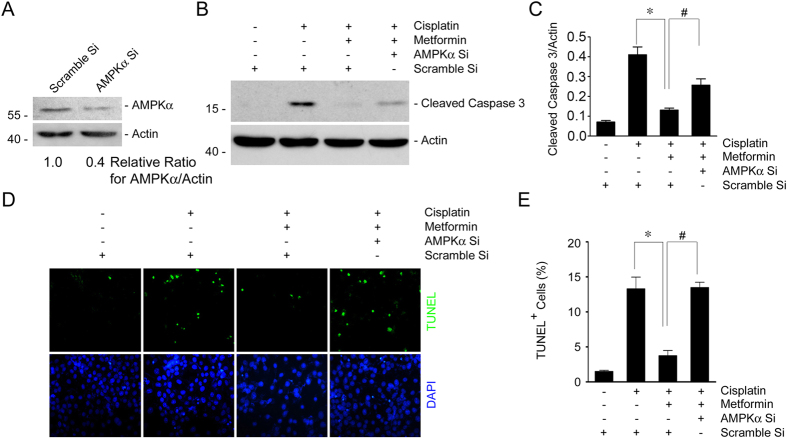
Inhibition of AMPKα with siRNA antagonizes the protective effect for metformin in cisplatin-induced cell apoptosis. (**A**) Western blot assay showing the downregulation of AMPKα expression in NRK-52E cells transfected with AMPKα siRNA. The gels were run under the same experimental conditions. (**B**) Western blot assay showing the caspase 3 cleavage after cisplatin treatment in NRK-52E cells. The gels were run under the same experimental conditions. (**C**) The graphs showing the semi-quantitative analysis of the abundance for cleaved caspase 3 protein in NRK-52E cells within different groups. *P < 0.05 compared to the cells treated with cisplatin alone (n = 3); ^#^*P* < 0.05 compared to the cells treated with cisplatin plus metformin (n = 3). (**D**) Representative micrographs showing the results for TUNEL staining among different groups as indicated. (**E**) The graphs showing the quantitative determination of TUNEL-positive cells among different groups. Data are presented as the percentage of the TUNEL-positive cells. *P < 0.05 compared to the cells treated with cisplatin alone (n = 3); ^#^*P* < 0.05 compared to the cells treated with cisplatin plus metformin (n = 3).

Metformin could induce the phosphorylation of AMPKα in NRK-52E cells. We then want to explore the role of AMPKα activation in metformin-promoted tubular epithelial cell survival. Small interference RNA was used to down regulate AMPKα expression in NRK-52E cells. Western blotting assay showed that AMPKα siRNA transfection could downregulate about 60% of AMPKα protein expression compared to the cells transfected with scramble siRNA (Fig. 8A). NRK-52E cells transfected with scramble or AMPKα siRNA were treated with cisplatin w/o metformin for 12 hours. Western blotting assay showed that metformin could reduce cisplatin-stimulated caspase 3 cleavage, while AMPKα siRNA transfection could partly restore the reduction of caspase 3 cleavage in cells treated with metformin plus cisplatin (Fig. 8B,C). TUNEL staining further confirmed the results of western blotting assay (Fig. 8D,E). Together, these results suggest that metformin protects against cisplatin-induced NRK-52E cell apoptosis, at least in part, through AMPKα activation.

## Discussion

In this study, we pre-administrated the mice with metformin and found that metformin could efficiently protect against cisplatin-induced tubular cell apoptosis and AKI, which is partly through the activation of AMPKα and the induction of autophagy in tubular cells.

Metformin has been widely used in patients with type 2 diabetes for its glucose-lowering effect through the inhibition of liver gluconeogenesis as well as increasing insulin-mediated glucose uptake in the skeletal muscle^27^. Although it is reported that metformin may rescue tumor cells from cisplatin-induced death through Akt-dependent mechanism^36^, multiple viewpoints have looked into the pleiotrophic potential of metformin in various types of disease, especially focusing on a notable reduction of cancer-related mortality with attenuated cancer progression^28^,^29^,^30^,^31^,^32^. Previous study found that metformin is protective in the gentamicin-induced nephrotoxicity^37^. In the present study, we demonstrated that pretreatment of metformin could prevent cisplatin-induced acute kidney injury in mice. However, Sahu *et al*. reported that metformin could attenuate the increase in malondialdehyde and tROS generation and restore the decrease in both enzymatic and non-enzymatic antioxidants, but couldn’t affect cisplatin-induced kidney abnormality in terms of renal functional and histological changes in rats^38^. We think the difference as to the species, the administrating route as well as the dosage for metformin we used may account for the discrepancy between ours and Sahu’s^38^.

Many studies demonstrated that tubular cell apoptosis is one of the major mechanisms leading to cisplatin-induced acute kidney injury^5^,^39^. In this study, metformin could inhibit cisplatin-induced tubular cells apoptosis in both cultured kidney tubular cells and in mouse model. In addition, excessive inflammatory cell infiltration in the kidneys is also involved into tubular collapse and kidney failure^40^. Recent studies found that metformin may inhibit TNF-α-induced inflammatory signaling activation and iNOS expression in lung tissue of obese mice^41^. In this study, neutrophil and macrophage infiltration in the kidneys were largely diminished by metformin administration. Based on these observations, it may be concluded that metformin ameliorates cisplatin-induced nephrotoxicity through inhibiting both tubular cell death and interstitial inflammation.

Autophagy is crucially involved in various biological processes such as cell growth, development and homeostasis via maintaining a balance between the synthesis, degradation, and subsequent recycling of cellular components^7^. Autophagy is rapidly induced in the kidney tissue after cisplatin treatment and such induction is protective against cisplatin induced tubular cell apoptosis and kidney injury. Inhibition of autophagy with pharmacological inhibitors as well as autophagy gene deletion strategy enhances cisplatin-induced tubular cell death and kidney injury^12^,^13^,^14^,^42^. Thus, autophagy induction is able to attenuate cisplatin-induced renal injury through antagonizing apoptotic cell death. In this study, we found that metformin could stimulate autophagic activity in both cultured tubular cells and mice received cisplatin treatment. Additionally, 3-methyladenine, an autophagy inhibitor^43^, could antagonize the protective effect for metformin in cisplatin-induced tubular cell death, suggesting that the autophagy induction is required for metformin in protecting against cisplatin induced tubular cell death.

AMPK, the major target of metformin, acts as the sensor of cellular energy supplies and controls protein synthesis, apoptosis and autophagy^18^,^19^. Previous studies showed that the activation of AMPK may inhibit mTORC1 signaling and stimulate autophagy^42^. Xie *et al*. reported that metformin can protect against cardiac dysfunction following myocardial ischemia through activating AMPK and cardiac autophagy^44^. In this study, we found that metformin could enhance AMPKα phosphorylation in both kidneys from mice and cultured tubular cells in the presence of cisplatin. Additionally, down regulating AMPKα expression with small interfering RNA could partly antagonize the protective effect of metformin in cisplatin-induced tubular cell death, which suggests that AMPKα activation is crucial for mediating the protective effect of metformin in cisplatin-induced nephrotoxity. However, except for AMPKα activation, some other mechanisms may exist in mediating the protective effect for metformin in cisplatin-induced tubular cell death, which needs more investigation.

In both prospective and retrospective studies, metformin has been reported to be associated with antineoplastic activity and decreased burden of many types of tumor^45^,^46^,^47^,^48^. Since cisplatin can reduce tumor mass through inducing DNA damage and apoptosis, it may be argued that metformin may antagonize the anti-tumor effect of cisplatin if used as a combination therapy. Fortunately, a combination of metformin and cisplatin exhibits better anti-tumor effect in many types of cancer in both clinical practice and animal experiments via inhibiting angiogenesis, metastatic spread and helping to overcome tumor cisplatin resistance in ovarian cancer or non-small-cell lung cancer^49^,^50^. Based on those reports, it may be concluded that the combination of metformin with cisplatin is safe for patients suffering from solid tumors.

In conclusion, we demonstrated here that metformin is protective in cisplatin-induced tubular cell death and AKI, which is probably mediated by promoting AMPK activation and autophagy induction in tubular cells. This study may elicit a new therapeutic strategy for the patients suffering from cisplatin-induced nephrotoxicity.

## Methods

### Mice and animal Models

Male CD1 mice weighing approximately 20–25 g were acquired from the Specific Pathogen-Free (SPF) Laboratory Animal Center of Nanjing Medical University and maintained according to the guidelines of the Institutional Animal Care and Use Committee at Nanjing Medical University. To generate the cisplatin-induced AKI model, mice were injected with a single dose of cisplatin (20 mg/kg) intraperitoneally. To investigate the protective effect of metformin in cisplatin-induced AKI, metformin was administrated 3 days before cisplatin injection at dosages of 100 mg/kg and 200 mg/kg per day. Mice were sacrificed at day 2 after cisplatin injection. Blood and kidney samples were harvested for further analysis. All experiments were performed in accordance with the approved guidelines and regulations by the Animal Experimentation Ethics Committee at Nanjing Medical University. All experimental protocols were approved by the Animal Experimentation Ethics Committee at Nanjing Medical University.

### Serum BUN assay

Serum BUN was measured with the QuantiChrom Urea Assay kit (cat: DIUR-500, Hayward, CA) according to the manufacturer’s instructions.

### Cell culture and treatment

Normal Rat Kidney epithelial cells (NRK-52E) were obtained from ATCC (CRL-1571TM, Manassas, VA). Cells were cultured in Dulbecco’s modified Eagle’smedium-F12 medium supplemented with 10% fetal bovine serum (Invitrogen, Grand Island, NY). Cells were seeded on six-well culture plate and let the cells grow to 60–70% confluence in complete medium containing 10% FBS for 16 h, then changed to serum-free medium after washing twice with serum-free medium. Metformin (cat: 100-B-010-CF, Sigma-Aldrich, St Louis, MO) was added at the final concentration of 4 mM. AMPKα siRNA (Integrated Biotech Solutions, Shanghai, China) and GFP-LC3 expression plasmid provided by Wen-Xing Ding (The University of Kansas Medical Center, Kansas City, KS, USA) were transfected into NRK-52E cells using Lipofectamine 2000 reagent (Invitrogen, Grand Island, NY) according to the manufacturer’s instructions. NRK-52E cells were incubated with 20 μg/ml of cisplatin for 12 h to induce cell death.

### Histology and immunohistochemistry

Kidney samples were fixed in 10% neutraformaline, embedded in paraffin. Sections at 3 μm thickness were used for PAS staining. To determine kidney injury, as defined by tubular necrosis, cellular casts, and tubular injury, a semi-quantitative scoring method was used. Score 0 represents injury area less than 10%, whereas score 1,2,3, or 4 represent the injury involving 10–25%, 25–50%, 50–75% or >75% of the field, respectively. At least ten randomly chosen fields under the microscope (×400) were evaluated for each mouse, and an average score was calculated. For immunohistochemical staining, paraffin-embedded kidney sections were deparaffinized, hydrated, antigen-retrieved, and endogenous peroxidase activity was quenched by 3% H_2_O_2_. Sections were then blocked with 10% normal donkey serum, followed by incubation with anti-Ly-6b (cat: MCA771G, AbD Serotec, Raleigh, NC) overnight at 4 °C. After incubation with secondary antibody for 1 h, sections were incubated with ABC reagents for 1 h at room temperature before subjected to substrate 3-amino-9-ethylcarbazole or DAB (Vector Laboratories, 7dBurlingame, CA). Slides were viewed with a Nikon Eclipse 80i microscope equipped with a digital camera (DS-Ri1, Nikon, Shanghai, China).

### Immunofluorescent staining

Kidney cryosections at 3 μm thickness were fixed for 15 min in 4% paraformaldehyde, followed by permeabilization with 0.2% TritonX-100 in PBS for 5 min at RT. After blocking with 2% donkey serum for 60 min, the slides were immunostained with primary antibodies against LC3-β (cat: L7543, Sigma Aldrich, St Louis, MO), cleaved caspase3 (cat: 9664, Cell Signaling Technology, Beverly, MA) and F4/80 (cat: 14–4801, eBioscience, San Diego, CA). The slides were then stained with TRITC or FITC-conjugated secondary antibodies. Slides were viewed with a Nikon Eclipse 80i Epifluorescence microscope equipped with a digital camera.

### Terminal deoxynucleotidyl transferase–mediated dUTP nick-end labeling (TUNEL) staining

Apoptotic cell death was determined by using terminal deoxynucleotidyl transferase–mediated dUTP nick-end labeling staining using the Apoptosis Detection System (Promega, Madison, WI).

### Annexin V/PI staining and Flow cytometric analysis

Briefly, NRK-52E cells were seeded on 100 mm culture dishes to 60–70% confluence in complete medium containing 10% FBS for 16 h, then changed to serum-free medium after washing twice with serum-free medium. Metformin (cat: 100-B-010-CF, Sigma-Aldrich, St Louis, MO) was added (4 mM), 30 minutes later, followed by cisplatin (20 μg/ml) administration for 12 h. The cells were harvested, stained with annexin V-FITC/PI staining kit according to the manufacturer’s instruction (cat: APOAF, Sigma-Aldrich, St Louis, MO) and analyzed by flow cytometry to identify cell apoptosis (BD FACSCanto II, BD Biosciences, SanJose, CA). Annexin V^+^/PI^−^ cells were considered as early apoptotic cells, and Annexin V^+^/PI^+^ cells were considered as late apoptotic cells.

### Western blot analysis

Cultural NRK-52E cells were lysed in 1 × SDS sample buffer. The kidneys were lysed with RIPA solution containing 1% NP40, 0.1%SDS, 100 mg/ml PMSF, 1% protease inhibitor cocktail, and 1% phosphatase I and II inhibitor cocktail (Sigma, St Louis, MO) on ice. The supernatants were collected after centrifugation at 13,000 × g at 4 °C for 30 min. Protein concentration was determined by bicinchoninic acid protein assay. An equal amount of protein was loaded into 10% or 15% SDS-PAGE and transferred onto polyvinylidene difluoride membranes. The primary antibodies were as follows: anti-LC3-β (cat: L7543, Sigma Aldrich, St Louis, MO), anti-cleaved caspase3 (cat: 9664, Cell Signaling Technology, Beverly, MA), anti-β-actin (cat: sc-1616, Santa Cruz Biotechnology), anti-p-AMPKα (T172) (cat: 2535, Cell Signaling Technology, Beverly, MA), anti-AMPKα (cat: 5831, Cell Signaling Technology, Beverly, MA), anti-p-S6 (S235/236) (cat: 4857, Cell Signaling Technology, Beverly, MA). Quantification was performed by measuring the intensity of the signals with the aid of National Institutes of Health Image software package.

### Statistical analysis

All data examined are presented as mean ± s.e.m. Statistical analysis of the data was performed using the Sigma Stat software (Jandel Scientific Software, San Rafael, CA). Comparison among three or more groups was made using one-way ANOVA, followed by the Student-Newman-Keuls test. Comparison between two groups was made using Student’s t-test. *P* < 0.05 was considered statistically significant.

## Additional Information

**How to cite this article**: Li, J. *et al*. Metformin Protects Against Cisplatin-Induced Tubular Cell Apoptosis and Acute Kidney Injury via AMPKα-regulated Autophagy Induction. *Sci. Rep.* **6**, 23975; doi: 10.1038/srep23975 (2016).

## References

[b1] Siddik Z. H. Cisplatin: mode of cytotoxic action and molecular basis of resistance. Oncogene 22, 7265–79 (2003). 14576837 10.1038/sj.onc.1206933

[b2] Lameire N. H. . Acute kidney injury: an increasing global concern. Lancet 382, 170–9 (2013). 23727171 10.1016/S0140-6736(13)60647-9

[b3] Sanchez-Gonzalez P. D., Lopez-Hernandez F. J., Lopez-Novoa J. M. & Morales A. I. An integrative view of the pathophysiological events leading to cisplatin nephrotoxicity. Crit Rev Toxicol. 41, 803–21 (2011). 21838551 10.3109/10408444.2011.602662

[b4] Bonegio R. & Lieberthal W. Role of apoptosis in the pathogenesis of acute renal failure. Curr Opin Nephrol Hypertens 11, 301–8 (2002). 11981260 10.1097/00041552-200205000-00006

[b5] Pabla N. & Dong Z. Cisplatin nephrotoxicity: mechanisms and renoprotective strategies. Kidney Int 73, 994–1007 (2008). 18272962 10.1038/sj.ki.5002786

[b6] Gabbiani C., Magherini F., Modesti A. & Messori L. Proteomic and metallomic strategies for understanding the mode of action of anticancer metallodrugs. Anticancer Agents Med Chem. 10, 324–37 (2010). 20380635 10.2174/187152010791162315

[b7] Mizushima N., Ohsumi Y. & Yoshimori T. Autophagosome formation in mammalian cells. Cell Struct Funct 27, 421–9 (2002). 12576635 10.1247/csf.27.421

[b8] Periyasamy-Thandavan S. . Autophagy is cytoprotective during cisplatin injury of renal proximal tubular cells. Kidney Int 74, 631–40 (2008). 18509315 10.1038/ki.2008.214

[b9] Jiang M., Liu K., Luo J. & Dong Z. Autophagy is a renoprotective mechanism during *in vitro* hypoxia and *in vivo* ischemia-reperfusion injury. Am J Pathol. 176, 1181–92 (2010). 20075199 10.2353/ajpath.2010.090594 PMC2832141

[b10] Liu S. . Autophagy plays a critical role in kidney tubule maintenance, aging and ischemia-reperfusion injury. Autophagy 8, 826–37 (2012). 22617445 10.4161/auto.19419

[b11] Kimura T. . Autophagy protects the proximal tubule from degeneration and acute ischemic injury. J Am Soc Nephrol. 22, 902–13 (2011). 21493778 10.1681/ASN.2010070705 PMC3083312

[b12] Takahashi A. . Autophagy guards against cisplatin-induced acute kidney injury. Am J Pathol. 180, 517–25 (2012). 22265049 10.1016/j.ajpath.2011.11.001

[b13] Jiang M. . Autophagy in proximal tubules protects against acute kidney injury. Kidney Int 82, 1271–83 (2012). 22854643 10.1038/ki.2012.261 PMC3491167

[b14] Li J. . Rictor/mTORC2 protects against cisplatin-induced tubular cell death and acute kidney injury. Kidney Int 86, 86–102 (2014). 24451322 10.1038/ki.2013.559

[b15] Yamahara K. . Obesity-mediated autophagy insufficiency exacerbates proteinuria-induced tubulointerstitial lesions. J Am Soc Nephrol. 24, 1769–81 (2013). 24092929 10.1681/ASN.2012111080 PMC3810079

[b16] Grahammer F. . mTORC1 maintains renal tubular homeostasis and is essential in response to ischemic stress. Proc Natl Acad Sci USA 111, E2817–26 (2014). 24958889 10.1073/pnas.1402352111 PMC4103333

[b17] Li L., Wang Z. V., Hill J. A. & Lin F. New autophagy reporter mice reveal dynamics of proximal tubular autophagy. J Am Soc Nephrol 25, 305–15 (2014). 24179166 10.1681/ASN.2013040374 PMC3904563

[b18] Hardie D. G. AMPK: positive and negative regulation, and its role in whole-body energy homeostasis. Curr Opin Cell Biol. 33, 1–7 (2015). 25259783 10.1016/j.ceb.2014.09.004

[b19] Mihaylova M. M. & Shaw R. J. The AMPK signalling pathway coordinates cell growth, autophagy and metabolism. Nat Cell Biol. 13, 1016–23 (2011). 21892142 10.1038/ncb2329 PMC3249400

[b20] Miller R. A. & Birnbaum M. J. An energetic tale of AMPK-independent effects of metformin. J Clin Invest 120, 2267–70 (2010). 20577046 10.1172/JCI43661 PMC2898617

[b21] Ouyang J., Parakhia R. A. & Ochs R. S. Metformin activates AMP kinase through inhibition of AMP deaminase. J Biol Chem. 286, 1–11 (2011). 21059655 10.1074/jbc.M110.121806 PMC3012963

[b22] Tripathi D. N. . Reactive nitrogen species regulate autophagy through ATM-AMPK-TSC2-mediated suppression of mTORC1. Proc Natl Acad Sci USA 110, E2950–7 (2013). 23878245 10.1073/pnas.1307736110 PMC3740898

[b23] Hardie D. G., Ross F. A. & Hawley S. A. AMPK: a nutrient and energy sensor that maintains energy homeostasis. Nat Rev Mol Cell Biol 13, 251–62 (2012). 22436748 10.1038/nrm3311 PMC5726489

[b24] Decleves A. E., Sharma K. & Satriano J. Beneficial Effects of AMP-Activated Protein Kinase Agonists in Kidney Ischemia-Reperfusion: Autophagy and Cellular Stress Markers. Nephron Exp Nephrol. (2014). 10.1159/000368932 PMC4458239 25503637

[b25] He L. . Metformin and insulin suppress hepatic gluconeogenesis through phosphorylation of CREB binding protein. Cell 137, 635–46 (2009). 19450513 10.1016/j.cell.2009.03.016 PMC2775562

[b26] Stratton I. M. . Association of glycaemia with macrovascular and microvascular complications of type 2 diabetes (UKPDS 35): prospective observational study. BMJ 321, 405–12 (2000). 10938048 10.1136/bmj.321.7258.405 PMC27454

[b27] Viollet B. . Cellular and molecular mechanisms of metformin: an overview. Clin Sci (Lond) 122, 253–70 (2012). 22117616 10.1042/CS20110386 PMC3398862

[b28] Giovannucci E. . Diabetes and cancer: a consensus report. Diabetes Care 33, 1674–85 (2010). 20587728 10.2337/dc10-0666 PMC2890380

[b29] Libby G. . New users of metformin are at low risk of incident cancer: a cohort study among people with type 2 diabetes. Diabetes Care 32, 1620–5 (2009). 19564453 10.2337/dc08-2175 PMC2732153

[b30] Schneider M. B. . Prevention of pancreatic cancer induction in hamsters by metformin. Gastroenterology 120, 1263–70 (2001). 11266389 10.1053/gast.2001.23258

[b31] Ohno T. . Metformin suppresses diethylnitrosamine-induced liver tumorigenesis in obese and diabetic C57BL/KsJ-+Leprdb/+Leprdb mice. Plos One 10, e0124081 (2015). 25879666 10.1371/journal.pone.0124081 PMC4399835

[b32] Chen H. P. . Metformin decreases hepatocellular carcinoma risk in a dose-dependent manner: population-based and *in vitro* studies. Gut 62, 606–15 (2013). 22773548 10.1136/gutjnl-2011-301708

[b33] Zhou G. . Role of AMP-activated protein kinase in mechanism of metformin action. J Clin Invest 108, 1167–74 (2001). 11602624 10.1172/JCI13505 PMC209533

[b34] Dowling R. J., Zakikhani M., Fantus I. G., Pollak M. & Sonenberg N. Metformin inhibits mammalian target of rapamycin-dependent translation initiation in breast cancer cells. Cancer Res 67, 10804–12 (2007). 18006825 10.1158/0008-5472.CAN-07-2310

[b35] Mao-Ying Q. L. . The anti-diabetic drug metformin protects against chemotherapy-induced peripheral neuropathy in a mouse model. Plos One 9, e100701 (2014). 24955774 10.1371/journal.pone.0100701 PMC4067328

[b36] Janjetovic K. . Metformin reduces cisplatin-mediated apoptotic death of cancer cells through AMPK-independent activation of Akt. Eur J Pharmacol. 651, 41–50 (2011). 21114978 10.1016/j.ejphar.2010.11.005

[b37] Morales A. I. . Metformin prevents experimental gentamicin-induced nephropathy by a mitochondria-dependent pathway. Kidney Int 77, 861–9 (2010). 20164825 10.1038/ki.2010.11

[b38] Sahu B. D., Kuncha M., Putcha U. K. & Sistla R. Effect of metformin against cisplatin induced acute renal injury in rats: a biochemical and histoarchitectural evaluation. Exp Toxicol Pathol. 65, 933–40 (2013). 23395153 10.1016/j.etp.2013.01.007

[b39] Kaushal G. P., Kaushal V., Hong X. & Shah S. V. Role and regulation of activation of caspases in cisplatin-induced injury to renal tubular epithelial cells. Kidney Int 60, 1726–36 (2001). 11703590 10.1046/j.1523-1755.2001.00026.x

[b40] Kinsey G. R., Li L. & Okusa M. D. Inflammation in acute kidney injury. Nephron Exp Nephrol. 109, e102–7 (2008). 18802372 10.1159/000142934 PMC2614446

[b41] Calixto M. C. . Metformin attenuates the exacerbation of the allergic eosinophilic inflammation in high fat-diet-induced obesity in mice. Plos One 8, e76786 (2013). 24204674 10.1371/journal.pone.0076786 PMC3811997

[b42] He L., Livingston M. J. & Dong Z. Autophagy in acute kidney injury and repair. Nephron Clin Pract. 127, 56–60 (2014). 25343822 10.1159/000363677 PMC4274769

[b43] Wu Y. T. . Dual role of 3-methyladenine in modulation of autophagy via different temporal patterns of inhibition on class I and III phosphoinositide 3-kinase. J Biol Chem. 285, 10850–61 (2010). 20123989 10.1074/jbc.M109.080796 PMC2856291

[b44] Xie Z. . Improvement of cardiac functions by chronic metformin treatment is associated with enhanced cardiac autophagy in diabetic OVE26 mice. Diabetes 60, 1770–8 (2011). 21562078 10.2337/db10-0351 PMC3114402

[b45] Kordes S. . Metformin in patients with advanced pancreatic cancer: a double-blind, randomised, placebo-controlled phase 2 trial. Lancet Oncol. 16, 839–47 (2015). 26067687 10.1016/S1470-2045(15)00027-3

[b46] Greenhill C. Gastric cancer. Metformin improves survival and recurrence rate in patients with diabetes and gastric cancer. Nat Rev Gastroenterol Hepatol. 12, 124 (2015). 25623201 10.1038/nrgastro.2015.9

[b47] Lin J. J. . Survival of patients with stage IV lung cancer with diabetes treated with metformin. Am J Respir Crit Care Med. 191, 448–54 (2015). 25522257 10.1164/rccm.201407-1395OC PMC4351595

[b48] Margel D. . Metformin use and all-cause and prostate cancer-specific mortality among men with diabetes. J Clin Oncol. 31, 3069–75 (2013). 23918942 10.1200/JCO.2012.46.7043

[b49] Lin C. C. . Metformin enhances cisplatin cytotoxicity by suppressing signal transducer and activator of transcription-3 activity independently of the liver kinase B1-AMP-activated protein kinase pathway. Am J Respir Cell Mol Biol. 49, 241–50 (2013). 23526220 10.1165/rcmb.2012-0244OC

[b50] Rattan R., Graham R. P., Maguire J. L., Giri S. & Shridhar V. Metformin suppresses ovarian cancer growth and metastasis with enhancement of cisplatin cytotoxicity *in vivo*. Neoplasia 13, 483–91 (2011). 21532889 10.1593/neo.11148 PMC3084625

